# Impact of fashion braces on oral health related quality of life: a web-based cross-sectional study

**DOI:** 10.1186/s12903-020-01224-1

**Published:** 2020-08-26

**Authors:** Zaki Hakami, Hye Soo Chung, Seham Moafa, Hadia Nasser, Hajar Sowadi, Safeena Saheb, Ahmed M. Bokhari, Nina K. Anderson

**Affiliations:** 1 Division of Orthodontics, Department of Preventive Dental Sciences, College of Dentistry, Jazan University, Jazan, Saudi Arabia; 2 Harvard School of Dental Medicine, Harvard University, Boston, USA; 3 College of Dentistry, Jazan University, Jazan, Saudi Arabia; 4 Division of Dental Public Health, Department of Preventive Dental Sciences, College of Dentistry, Jazan University, Jazan, Saudi Arabia; 5 Department of Orthodontics and Pediatric Medicine, Stony Brook School of Dental Medicine, Stony Brook, USA

**Keywords:** Fashion braces, Fashion brackets, Fake braces, Fake brackets, Oral health, Quality of life

## Abstract

**Background:**

Orthodontic braces have become symbols of wealth and fashion accessories in some parts of the world. However, there is a scarcity of information about the effects of fashion braces on various aspects of quality of life. Therefore, our aim was to investigate the effects of fashion braces on oral health related quality of life (OHRQoL).

**Methods:**

A cross-sectional study was carried out with data collection from a Google form questionnaire distributed in Saudi Arabia via various forms of social media over a period of 4 months. OHRQoL was assessed using the validated Arabic version of the Oral Health Impact Profile-14 (OHIP-14) questionnaire. The fashion braces group included respondents who had braces installed for fashion purposes only. Therapeutic braces group included subjects who were wearing braces to treat any malocclusion problems. Control group included subjects who did not have any kind of braces. People who had previously completed orthodontic treatments were excluded from the study. The negative impacts were divided into seven domains and a total OHIP score was calculated. Statistical analyses and data illustration were performed with SPSS v25 (IBM, NY).

**Results:**

A total of 1141 people voluntarily participated in the study. More than 60% of the participants were in the control group while 33.7% had conventional braces for therapeutic reasons and 3.4% had fashion braces. Sociodemographic distributions varied among the groups, with the majority of the fashion braces group having education below the university level and family incomes less than average. There were significant group differences in OHIP domains. Physical pain was the most frequently reported complaint by all subjects and was the highest in the therapeutic braces group. People with therapeutic braces reported significantly higher functional limitation and physical disability than the controls. Fashion braces group reported significantly lower psychological discomfort and disability, social disability and handicap compared to control groups.

**Conclusions:**

The illustrated effects of fashion braces on OHRQoL suggest the need to study the role of social media and educate the public on the use of braces to minimize the negative effects experienced by individuals.

## Background

Optimum oral health is an essential part of the overall health of an individual. Poor oral health can significantly lower a person’s quality of life by negatively affecting their functions such as eating, speaking and smiling as well as their social life [1]. It is therefore necessary to assess the oral health related quality of life (OHRQoL) during treatment as well as after treatment [2]. OHRQoL is a multidimensional construct that includes a subjective evaluation of an individual’s oral health, functional wellbeing, emotional wellbeing, expectations and satisfaction [3]. OHRQoL is worth examining since orthodontic treatment has been found to have both advantageous and adverse effects associated with the execution of treatment. For example, some studies found that when compared with pretreatment, a patient’s OHROoL is frequently worse during orthodontic treatment with oral symptoms and functional limitations, but it is better in other aspects such as emotional wellbeing [4, 5].

Although orthodontic treatment has been considered successful for occlusal functions and esthetic demands [6], many patients hesitate to seek the appropriate treatments because of the high cost of fixed orthodontic appliances. The high demand for braces due to increasing aesthetic concerns of the public, long-term commitment necessary to correct the malocclusion, and orthodontic student debt load, are some of many factors that lead to the high costs of orthodontic treatments [7]. Due to the high cost and the belief that orthodontic treatment is an elective luxury, braces have become a sign of financial prosperity in some South Asian countries [7]. It has become a symbol of status, wealth and style. Due to this trend, a type of fixed braces called “fashion braces” that have unique or esthetic bracket designs have become a popular option for many people. These braces serve as fashion statements without any therapeutic effects of conventional braces. Fashion braces are largely provided by dental professionals, however, cheap braces or braces like jewelry are also now available at a much lower cost, which can be fitted in a dental office or even at home [8].

These fashion braces may change OHRQoL in a positive or negative way. Nevertheless, there is a scarcity of knowledge in that regard as little, if any, research has been conducted. Therefore, the objective of this study was to investigate the effects of fashion braces on OHRQoL in comparison with conventional therapeutic braces as well as control group without any type of orthodontic appliances.

## Methods

Ethical approval (# CODJU-18081) was reviewed and approved by the ethical committee of the scientific research unit, College of Dentistry, Jazan University before commencement of the research.

A cross-sectional study was conducted to investigate the relationship between type of braces patients wear and OHRQoL. Participants were classified into three groups – therapeutic braces, fashion braces and control groups. Therapeutic braces group included subjects who were wearing fixed braces for orthodontic treatment purposes. Fashion braces group included subjects who were wearing fixed braces for fashion only. The control group included subjects who were not wearing any kind of braces. Exclusion criteria included those subjects who reported having completed any type of orthodontic treatment.

A snowball sampling strategy was utilized. The online survey was first disseminated to university students and they were encouraged to pass it on to others. After pre-testing, a google form questionnaire in Arabic language was distributed to residents in Saudi Arabia via social media including WhatsApp, Twitter, Facebook, Instagram and Snapchat for 4 months from November 2018 to February 2019. Before answering the questions, written informed consent was obtained and parental consent was required for participants under the age of 18. Each participant was asked to provide sociodemographic information such as age, gender, educational level, family income, smoking status and frequency of brushing, as well as, noticing teeth color changes. Average family income was set as ten thousand Saudi Arabian Riyal (SAR) per month according to General Authority of Statistics in Saudi Arabia. Participants wearing fashion braces were asked to provide their background information, including cost of the braces, location of braces placement, classification of braces provider, opinions on the price, whether they visit their providers periodically and frequency of dental visits. Full text of the questionnaire can be found in the Additional file 1.

OHRQoL was assessed using the validated Arabic version of OHIP-14 questionnaire [9]. Participants were asked to rate the frequency they had experienced various negative impacts regarding oral health during the previous 12 months. The items included: had problem pronouncing words, felt sense of taste worsened, had painful aching in mouth, found it uncomfortable to eat food, have been self-conscious, felt tense, had an unsatisfactory diet, had to interrupt meals, found it difficult to relax, have been a bit embarrassed, have been irritable with people, had difficulty doing useful jobs, felt life in general less satisfactory, and have been unable to function. Responses were recorded on a 5-point Likert-type scale (0 – never, 1 – hardly ever, 2 – occasionally, 3 – fairly often and 4 – very often).

After the participants’ responses were collected, the OHIP-14 questions were divided into seven domains – functional limitation, physical pain, psychological discomfort, physical disability, psychological disability, social disability and handicap. Following the OHIP protocol, each domain included two different daily activity questions from the questionnaire [10]. The domain score was calculated by adding up the numerical response of each question. Then, total OHIP score was obtained for each participant by adding up the scores of all seven domains. Overall, the higher the domain score, the poorer the OHRQoL.

### Statistical analysis

Statistical analysis and data illustration were performed with SPSS Statistics (v. 25; IBM, NY).

Descriptive statistics including mean and percentage distribution was calculated. In order to compare the participants’ profile according to types of braces, Chi-square analyses were performed. All variables were tested for normality using the Shapiro–Wilk test to identify any differences among the three groups. According to the normality of the data, non-parametric Mann-Whitney U test, Kruskal Wallis test with post-hoc Dunn’s Bonferroni test were carried out to assess the significant differences in the seven domains among gender, educational levels, family income and type of braces. Finally, Spearman correlation and logistic regression analyses were conducted to evaluate the association among the variables. The *p*-value was set at 0.05 for all tests.

## Results

### Sociodemographic distribution of the participants

A total of 1141 people participated in the questionnaire; mean age was 26.9 years ±7.46 and 58.1% were female. The majority of the participants (62.9% (*n* = 718)) were in the control group, whereas 33.7% (*n* = 384) wore orthodontic braces for therapeutic purposes and 3.4% (*n* = 39) wore orthodontic braces for fashion purposes.

In terms of educational level, 75.1% (*n* = 857) of participants had university-level, 20.7% (*n* = 236) high school-level, 3.6% (*n* = 41) intermediate-level and 0.6% (*n* = 7) elementary-level education. The majority of the participants in the fashion braces group (71.8% (*n* = 28)) had education below the university level. On the other hand, the majority of participants in the therapeutic braces group and the control group had the university level of education (74.3% (*n* = 293) and 77.0% (*n* = 553), respectively).

The participants were similarly divided into three family income groups, 35.3% (*n* = 403) earning more than average, 32.4% (*n* = 370) less than average and 32.3% (*n* = 368) average. The majority of the participants in the fashion braces group had family income of less than average. The distribution of family income in the therapeutic braces group was close to each other. The family income in the control group was 37.7% (*n* = 271) earning more than average, 29.5% (*n* = 212) less than average and 32.7% (*n* = 235) average. Significant differences were found on gender, educational level and family income among the groups. See Table 1 for distribution of participants’ background.

**Table 1:** Distribution of subjects according to background profiles such as gender, type of braces, educational level, family income, smoking/shesha, frequency of brushing per day and noticing teeth color changes

Characteristics	Total N (%)	Fashion braces group	Therapeutic braces group	Control group	P value ^a^
Gender					
Male	478 (41.9%)	18 (46.2%)	126 (32.8%)	334 (46.5%)	<.001
Female	663 (58.1%)	21 (53.8%)	258 (67.2%)	384 (53.5%)	
Educational level					<.001
Elementary	7 (0.6%)	2 (5.1%)	2 (0.5%)	3 (0.4%)	
Intermediate	41 (3.6%)	10 (25.6%)	9 (2.3%)	22 (3.1%)	
High school	236 (20.7%)	16 (41.0%)	80 (20.8%)	140 (19.5%)	
University	857 (75.1%)	11 (28.2%)	293 (74.3%)	553 (77.0%)	
Family income					.001
Less than average	370 (32.4%)	24 (61.5%)	134 (34.9%)	212 (29.5%)	
Average (SAR 10,000)	368 (32.3%)	7 (17.9%)	126 (32.8%)	235 (32.7%)	
More than average	403 (35.3%)	8 (20.5%)	124 (32.3%)	271 (37.7%)	.005
Smoking / shesha					
Yes	219 (19,1%)	11 (26.8%)	56 (14.2%)	152 (21.2%)	
No	914 (80.1%)	24 (63.4%)	327 (85.5%)	563 (78.4%)	
Unknown	8 (0.7%)	4 (9.8%)	1 (0.3%)	3 (0.4%)	
Frequency of brushing per day					<.001
Once	451 (39.5%)	7 (17.9%)	100 (26.0%)	344 (47.9%)	
Twice	477 (41.8%)	22 (56.4%)	186 (48.4%)	269 (37.5%)	
More than twice	141 (12.4%)	4 (10.3%)	83 (21.6%)	55 (7.7%)	
Do not brush	60 (5.3%)	2 (5.1%)	13 (3.4%)	45 (6.3%)	
Other	12 (1.1%)	4 (10.3%)	3 (0.8%)	5 (0.7%)	
Noticing teeth color changes					.57
Yes	588 (51.5%)	16 (41.0%)	200 (52.1%)	372 (51.8%)	
No	300 (26.3%)	14 (35.9%)	95 (24.7%)	191 (26.5%)	
Maybe	253 (22.2%)	9 (23.1%)	89 (23.2%)	155 (21.6%)	

^a^ Chi square test

The majority of the participants (93.3% (*n* = 36)) had their fashion braces placed in private clinics. 43.3% (*n* = 17) of the fashion braces were placed by orthodontists, 20.0% (*n* = 8) by general dentists, and 3.3% (n = 1) by dental assistants; 33.3% (*n* = 13) of the participants with fashion braces were not aware of the specific classification of who placed the braces. 40.0% (*n* = 16) of participants with fashion braces perceived the cost of the fashion braces as suitable while 23.3% (*n* = 9) perceived it as unsuitable (expensive). The majority of the participants in the fashion braces group (66.7% (*n* = 26)) reported visiting their providers periodically; every month or every 4 months (43.3% (*n* = 17) and 36.7% (*n* = 14), respectively). Results are summarized in Table 2.

**Table 2:** Distribution of subjects with fashion braces according to where the braces were placed, who placed the braces, what subjects think about the cost of braces, whether subjects visit their providers periodically and how often subjects visit their providers

Background information	Distribution
Cost of braces	1068.1 ± 1488.2 SAR
Location of braces placement	
Government clinics	6.7% (n = 3)
Private clinics	93.3% (n = 36)
By myself at home	0.0%
Classification of braces provider	
Orthodontist	43.3% (n = 17)
General dentist	20.0% (n = 8)
Dental assistant	3.3% (*n* = 1)
Do not know	33.3% (n = 13)
Subjects’ opinions on the price of face braces	
Suitable (cheap)	40.0% (n = 16)
Somewhat suitable (average)	36.7% (n = 14)
Unsuitable (expensive)	23.3% (n = 9)
Whether subjects visit their providers periodically	
Yes	33.3% (n = 13)
No	66.7% (n = 26)
Frequency of visits	
Every month	43.3% (n = 17)
Every two months	6.7% (n = 3)
Every three months	13.3% (n = 5)
Every four months	36.7% (n = 14)

### Impact of fashion braces on OHRQoL

Overall, the ANOVA for total OHIP scores and all subscale scores of OHIP domains showed significant differences among the three groups (Table 3). Participants wearing fashion braces reported less total OHIP score compared to the control group.

**Table 3:** Mean and standard deviation of OHIP domain scores for fashion braces, therapeutic braces and control groups as well as pairs of groups that showed significant differences in the OHIP domain scores

OHIP Domains	Fashion braces (F) Mean (SD)	Therapeutic braces (T) Mean (SD)	Control (C) Mean (SD)	Significant Pairs
Physical pain	2.97 (2.29)	4.39 (1.71)	3.02 (1.89)	T vs. C; F vs. T
Functional limitation	1.69 (2.14)	1.43 (1.53)	1.02 (1.46)	T vs. C
Physical disability	2.00 (2.50)	2.36 (1.93)	1.39 (1.68)	T vs. C
Psychological discomfort	1.03 (1.78)	1.58 (1.83)	2.56 (2.30)	T vs. C; F vs. C
Psychological disability	1.31 (2.07)	1.70 (1.58)	2.05 (1.88)	T vs. C; F vs. C
Social disability	0.97 (1.86)	0.99 (1.41)	1.50 (1.78)	T vs. C; F vs. C
Handicap	0.41 (1.43)	0.79 (1.27)	1.18 (1.64)	T vs. C; F vs. C; F vs. T
Total OHIP	10.38 (10.52)	13.25 (7.84)	12.71 (9.28)	F vs. T

Physical pain was the most frequently reported complaints among all participants. Participants wearing therapeutic braces reported significantly greater physical pain than those with fashion braces or the control group. On the other hand, participants wearing fashion braces showed similar level of physical pain to the control group. Participants with therapeutic braces reported significantly higher levels of functional limitation and physical disability when compared to the participants in the control group.

As compared to participants with fashion braces or therapeutic braces, the control participants had significantly higher psychological discomfort, psychological disability, social disability and handicap domains. Furthermore, the average scores for handicap domain in participants with therapeutic braces was significantly higher than those with fashion braces.

### Factors influencing OHRQoL

Age, gender, educational level and family income showed significant differences and significant correlations in OHIP as shown in Table 4. Weak negative correlations were observed between age and physical pain, psychological disability and total OHIP. Females had higher physical pain and physical disability than males. Educational level showed significant differences in functional limitation with participants in high school level reporting higher mean functional limitation scores than those in the university level or above. Educational level showed negative correlation with functional limitation and physical pain.

**Table 4:** Results of Spearman Correlation tests for relationship between OHIP domains and age, gender, educational level, family income, smoking / shesha, frequency of brushing, noticing teeth color changes, noticing teeth movement after braces and noticing teeth color changes because of braces. Only statistically significant correlation coefficient (r) and *p*-values (*p* < 0.05) are shown

		Functional limitation	Physical pain	Psychological discomfort	Physical disability	Psychological disability	Social disability	Handicap	Total OHIP
Age	R		−0.08						
	P value		0.01						
Educational level	R	−0.08							
	P-value	0.01							
Family income	R		−0.07		−0.10	−0.08	− 0.06	− 0.10	−0.09
	*P*-value		0.01		0.001	0.01	0.05	0.001	0.002
Smoking / shesha	R								
	P-value								
Frequency of brushing	R	0.09	0.07						
	P-value	0.002	0.03						
Noticing teeth color changes	R	−0.12	−0.13	−0.10	−0.11	−0.15	−0.14	−0.11	−0.19
	P-value	0.001	0.001	0.001	0.001	0.001	0.001	0.001	0.001
Noticing teeth color changes because of braces	R			0.17					
	P-value			0.02					

Participants with less family income group had higher mean scores in all OHIP domains and total OHIP compared to participants with higher family incomes. Moreover, participants with average family income reported higher average score in physical disability and handicap domains, as well as, total OHIP than participants with high family income. Family income correlated negatively with all OHIP domains and total OHIP except functional limitation, psychological discomfort and social disability (Table 4).

Table 5 presents the results of logistic regression. The regression results of age, gender, educational level and family income differed from the Spearman correlation tests. Aging tended to be associated with a higher likelihood of having psychological disability and handicap (0.89 and 0.84, respectively). Males were 0.2 times more likely to have social disability than females and participants with secondary school education were more likely to experience social disability. Participants from high incomes experienced 3.2 times more psychological discomfort than those from average and low-income family (Table 5).

**Table 5:** Association between OHIP domains with type of braces, demographics and oral health habits. Logistic regression is statistically significant at <.05*

Independent variable		Functional limitation	Physical pain	Psychological discomfort	Physical disability	Psychological disability	Social disability	Handicap	OHIP
		*Exp*(*β*) (CI 95%)	*Exp*(*β*) (CI 95%)	*Exp*(*β*) (CI 95%)	*Exp*(*β*) (CI 95%)	*Exp*(*β*) (CI 95%)	*Exp*(*β*) (CI 95%)	*Exp*(*β*) (CI 95%)	*Exp*(*β*) (CI 95%)
Age		.97 (.90–1.05)	.99 (.92–1.06)	.95 (.89–1.02)	.98 (.93–.1.04)	.89 (.82–.96)*	.91 (.82–1.01)	.84 (.72–.98)*	.93 (.87–1.00)
Gender	Male	1.57 (.62–3.99)	1.08 (.35–3.34)	.57 (.22–1.46)	1.17 (.53–2.59)	.89 (.35–2.25)	.20 (.04–.87)*	.86 (.20–3.63)	.88 (.36–2.16)
	Female	Reference							
Educational level	Primary school	.23 (.02–2.39)	.80 (.13–4.90)	.66 (.10–4.56)	.78 (.17–3.59)	1.83 (.33–10.17)	.60 (.06–6.40)	1.04 (.06–17.63)	1.89 (.33–10.84)
	Secondary school	.63 (.23–1.70)	.65 (.22–1.93)	.87 (.33–2.30)	.86 (.41–1.81)	.64 (.24–1.68)	.11 (.02–.73)*	.21 (.03–1.35)	1.50 (.62–3.66)
	University	Reference							
Family income	Less than	1.20 (.48–3.00)	.69 (.24–1.98)	1.41(.58–3.45)	.86 (.41–1.81)	.72 (.30–1.70)	1.12 (.33–3.81)	.51 (.13–2.07)	.70 (.30–1.64)
	More than	1.36 (.52–3.60)	1.01(.32–3.16)	3.14 (1.27–7.78)*	1.23 (.55–2.74)	1.19 (.48–2.92)	1.51 (.45–5.14)	.82 (21–3.19)	1.40 (.58–3.39)
	Average	Reference							
Type of braces	Therapeutic	2.67 (.30–23.55)	4.99 (1.09–22.91)*	.48 (.11–2.14)	1.20 (.45–.89)	.30 (.07–1.38)	.37 (.05–2.72)	.65 (.06–7.21)	.89 (.18–4.37)
	Fashion	2.94 (.22–38.92)	1.17 (.17–8.08)	.37 (.05–3.02)	2.59 (.40–16.89)	.30 (.04–2.31)	.99 (.06–15.76)	.43 (.01–14.56)	.25 (.25–2.56)
	Control	Reference							
Tooth color change	Yes	1.09 (.39–2.91)	2.39 (.84–6.87)	2.57 (1.01–6.57)*	2.29 (1.02–5.12)*	3.86 (1.40–10.64)*	2.99 (.06–15.76)	5.03 (.87–28.97)	3.16 (1.18–8.44)*
	Maybe	2.40 (.79–7.22)	2.58 (.70–9.43)	.81 (.26–2.61)	2.72 (1.06–6.97)*	2.08 (.66–6.55)	1.02 (.18–6.01)	1.56 (.19–13.12)	3.13 (1.04–9.44)*
	No	Reference							
Tooth color change with braces	Yes	1.42 (.64–3.24)	.55 (.23–1.44)	.54 (.26–1.13)	1.16 (.61–2.22)	1.71 (.80–3.54)	.67 (.24–1.83)	.81 (.26–2.49)	1.28 (.62–2.66)
	No	Reference							
Smoking Hookah	Yes	1.73 (.58–5.19)	.88 (.23–3.34)	1.21 (.41–3.56)	.51 (.33–1.56)	.81 (.33–2.35)	**6.44 (1.66–25.04**)*	1.25 (.28–5.57)	.66 (.22–1.97)
	No	Reference							
Tooth brushing	Twice	.63 (.25–1.55)	1.07 (.36–3.34)	.50 (.21–1.16)	.72 (.33–1.56)	.39 (.17–.90)*	.76 (.23–2.59)	.55 (.16–1.93)	.57 (.24–1.34)
	More than	.75 (.25–2.30)	1.06 (.36–3.17)	.36 (.12–1.06)	.62 (.27–1.73)	.22 (.07–.69)*	.92 (.21–3.97)	.13 (.01–1.33)	.39 (.13–1.34)
	Don’t brush	1.13 (.17–2.39)	1.08 (.10–12.12)	.001 (.001)	.62 (.11–3.47)	.24 (.02–2.44)	18.98 (1.80–199.74)*	.001 (.001)	1.09 (.19–6.20)
	Once	Reference							
		−2 Log = 168.74 *χ*^2^ = 12.42 P value = .65	− 2 Log = 136.25 *χ*^2^ = 16.26 *P* value = .37	− 2 Log = 179.04 *χ*^2^ = 29.37 P value = .01	− 2 Log = 225.59 *χ*^2^ = 10.48 P value = .80	− 2 Log = 181.29 *χ*^2^ = 28.80 *P* value = .02	− 2 Log =110.71 *χ*^2^ = 28.01 P value = .02	−2 Log =18.58 *χ*^2^ = 87.69 *P* value = .23	−2 Log = 186.57 *χ*^2^ = 20.10 P value = .17

Significant weak correlation were also observed between OHIP domains and smoking, frequency of brushing and noticing teeth color changes as shown in Table 4. Smoking was positively correlated with physical disability. Frequency of brushing showed weak positive correlations with functional limitation, physical pain and total OHIP. Noticing tooth color changes showed weak negative correlation to all domains, while noticing tooth color changes after tooth movement showed only significant negative correlation with psychological discomfort (Table 4).

An obvious effect of smoking, frequency of brushing and noticing teeth color changes was found by regression analysis (Table 5). Smoking and not brushing teeth had more association with social disability (6.4 and 18.9 times, respectively). Participants, who noticed teeth color changes, were two to three times more likely to report negative experiences in psychological discomfort, physical and psychological disability and total OHIP.

## Discussion

This study is considered the first in which the effect of fashion braces on OHRQoL has been evaluated. The results showed changes in OHRQoL with wearing braces for fashion only. Therapeutic and fashion braces had negative effects on functional limitation and physical disability, but was significantly greater for the therapeutic braces group. Nevertheless, fashion braces group reported significantly positive impacts on psychological and social domains.

Along with factors such as long treatment period and discomfort of the appliances, the high cost is one of the main concerns of orthodontic devices [7]. Currently, braces have become symbols of wealth and fashion accessories in some parts of Southeast Asia. The problem is that as fashion braces emerged as a statement of financial status, they have been advertised and sold through social media by unqualified personnel. In addition, the quality of the orthodontic brackets is low and cases of metal toxicity from those braces have been reported [11]. Investigating the impact of those non-therapeutic braces on quality of life is as important as studying uncovering potential health risks because a deeper understanding of the consequences of fashion braces will enable the patients to provide informed consent, have realistic expectations and provide a more accurate analysis of the cost and benefits of the devices [12]. This study, therefore, was conducted to assess the impact of fashion braces on OHRQoL with the hope of filling the gap of knowledge about fashion braces and educating the public about the effects of fashion braces.

Overall, the total OHIP scores showed no significant differences among the three groups of participants. Interestingly, both fashion and therapeutic braces groups showed lower levels of psychological discomfort, psychological disability, social disability and handicap than the control group. This is consistent with a previous study that showed decrease in all these criteria over time in individuals with conventional or self-ligating orthodontic devices [13]. Considering these OHIP domains consisted of questions regarding topics such as self-consciousness, embarrassment and dissatisfaction with life, this result suggests that wearing braces, therapeutic or fashion, could improve a person’s psychological and social well-being. These results concur with other studies underscoring the positive effects of orthodontic interventions on OHRQoL as well [4, 14]. Compared to the therapeutic braces group, the fashion braces group showed even lower levels of psychological discomfort and psychological disability, which means that fashion braces may indeed help people feel more confident and satisfied with themselves.

Despite the psychological and social benefits, both fashion and therapeutic braces groups showed greater functional limitation and physical disability than the control group, which is supported by a recent study that found a worsening trend in patients’ OHRQoL during orthodontic treatments [5]. In particular, fashion braces group showed significantly greater deterioration in these two criteria than the control group. In other words, people with fashion braces had more problems pronouncing words, felt sense of taste worsened, had unsatisfactory diets and had to interrupt meals due to their braces. People may prioritize the psychological benefits obtained by wearing fashion braces, but it is worth thinking about what the long-term physical and functional consequences will be.

The high cost of orthodontic treatment is one of the main considerations in terms of patient treatment decisions [7], so it is important to investigate the relationship between family income and OHRQoL. In this study, family income showed a negative correlation with all OHIP domains except for functional limitation, psychological discomfort and social disability. The low-income group showed higher OHIP domain level as well as total OHIP score when compared to high-income group. Simply put, low-income participants suffered from more problems than high-income participants. Even when high-income participants were compared to average-income participants, negative correlations between income and physical disability, handicap and total OHIP score were observed. High-income individuals reported fewer problems eating and functioning in life while wearing braces. This was consistent with a previous study conducted in Sweden that showed a significant association between poor OHRQoL and low income as well as having no economic resources [15]. These results suggest that high-income subjects generally had fewer problems with their braces perhaps because they had more resources to properly address the difficulties when needed.

Quality of life is a “dynamic construct” that is likely to change with age [16]. Our results showed that subjects’ OHRQoL was more likely to be impacted with aging. In our study, more than 70% of the subjects in the fashion braces group had education below the university level whereas more than 70% of the subjects in the therapeutic braces or control group had university level education. This conforms with the conclusion that the patients’ educational level had a direct influence on their knowledge and behavior regarding the main oral diseases and preventive measures [17]. It also supports a previous study in London that found low educational level has an independent negative impact on OHRQoL in older people [18]. People who have university level education may be more knowledgeable about the therapeutic effects of conventional braces and detrimental effects of fashion braces, so they may be more likely to avoid fashion braces. However, these results were only from a cross-sectional study and longitudinal studies are recommended.

In terms of family income, the participants of this study were somewhat equally divided between three groups of income less than average, average and more than average. The majority of participants in the fashion braces group had family income less than the national average in Saudi Arabia. This agrees with a previous study in which more than half the respondents came from a low-income family and the results showed that they thought fashion braces could be a cheaper and faster alternative to conventional orthodontic appliances [8]. Future studies with personal interviews would reveal more clear information about family income as it is associated with family size and consumption.

In this study, cross-sectional design was utilized as it is illegal to do a prospective study on fashion braces in Saudi Arabia and private dental centers were unwilling to participate. One main drawback of this study was that non-probability population sampling was used. Since the survey was distributed online as a Google form questionnaire via social media including What’s App, Twitter, Facebook, Instagram and Snapchat, not all residents in Saudi Arabia had an equal chance of being selected as participants in this study. For instance, the mean age of the participants who responded to the survey was 26.6 years old, which suggests that the younger population is more likely to use these types of social media to express their opinions. Moreover, there was not an equal representation of the three groups as more than 60% of the participants did not wear any braces while less than 5% of the participants had braces for fashion purposes. Since this survey was conducted as a pilot study with a small sample size, it is not safe to assume that the sample fully represents the target population. The results should be accepted with the realization that the study may lack representation of the population.

## Conclusion

Based on the preliminary results, this study showed changes in OHRQoL attributable to having fashion braces. Positive impacts were reported on psychological and social aspects of people wearing fashion braces. Social media is likely to play a major role in current society and should be included as a priority in future studies. Moreover, further clinical studies are needed to evaluate any adverse effects on dentition such as unwanted teeth movement, root resorption, caries and white spots lesions.

## Supplementary information

**Additional file 1.** “Questionnaire” contains English version, along with the original Arabic version of the questionnaire used for the survey in this research.

## Data Availability

The datasets used and/or analyzed during the current study are available from the corresponding author on reasonable request.

## Abbreviations

OHRQoL: Oral health related quality of life
OHIP-14: Oral Health Impact Profile-14
