# Supplementary material for: Impact of fashion braces on oral health related quality of life: a web-based cross-sectional study
Source: BMC Oral Health. 2020 Aug 26;20:236. doi: 10.1186/s12903-020-01224-1 (PMC7448975; doi:10.1186/s12903-020-01224-1)
Supplement: Supplementary file 1 — Additional file 1. “Questionnaire” contains English version, along with the original Arabic version of the questionnaire used for the survey in this research. [file 12903_2020_1224_MOESM1_ESM.docx]

**English Version Questionnaire**

Your Participation in this study is voluntarily, your answers will be used for research purposes only. Your parent consent is required if your age is below 18 years. 1- Yes 2- No.

- How old are you? ………. In years
- What is your gender?
  1. Male
  2. Female
- What is your educational level?
  1. Elementary School
  2. Intermediate School
  3. Secondary School
  4. University
  5. Other ………
- What is your family income?
  1. Average (10 thousands Saudi Arabian Riyal)
  2. Below than average
  3. Above than average
- Do you smoke cigarette or shesha?
  1. Yes
  2. No
- How many times do you brush your teeth per day?
  1. Once
  2. Twice
  3. More than that
  4. Less than that
  5. I don’t brush my teeth
  6. Other …………
- Have you noticed teeth color changes?
  1. Yes
  2. No
  3. May be
- Do you wear braces?
  1. Yes
  2. No
- What is the reason to wear braces?
  1. Aligning the teeth
  2. Increasing confidence
  3. Following the style or trend
  4. Influenced by social media
- Do you think wearing braces have added more confidence or beauty?
  1. Yes
  2. No
- Have you noticed any changes or movement in teeth after wearing the braces?
  1. Yes
  2. No
  3. May be
- Have you noticed any color changes in teeth because of the braces?
  1. Yes
  2. No
- What kind of braces are you wearing
  1. Therapeutic
  2. Fashion
- When have the braces been placed? ………………. Months
- Where is the location of braces placement?
  1. Government clinics
  2. Private clinics
  3. By myself at home
- What is the classification of braces provider?
  1. Orthodontist
  2. General dentist
  3. Dental assistant
  4. Don’t know
- What is your opinion about the prices of the braces?
  1. Suitable (cheap)
  2. Somewhat suitable (average)
  3. Unsuitable (expensive)
- Do you visit your provider periodically?
  1. Yes
  2. No
- If yes, how often do you visit your provider?
  1. Every month
  2. Every two months
  3. Every three months
  4. Every four months

**Questions to the fashion braces group about oral health related quality of life.**

**How frequently have you experienced negative impacts in these dimensions during the preceding 12 months?**

- Have you had trouble pronouncing any words because of the fashion braces?
  1. Never
  2. Hardly ever
  3. Occasionally
  4. Fair often
  5. Very often
- Have you felt that your sense of taste has worsened because of the fashion braces?
  1. Never
  2. Hardly ever
  3. Occasionally
  4. Fair often
  5. Very often
- Have you had painful aching in your mouth because of the fashion braces?
  1. Never
  2. Hardly ever
  3. Occasionally
  4. Fair often
  5. Very often
- Have you found it uncomfortable to eat any foods because of the fashion braces?
  1. Never
  2. Hardly ever
  3. Occasionally
  4. Fair often
  5. Very often
- Have you been self-conscious because of the fashion braces?
  1. Never
  2. Hardly ever
  3. Occasionally
  4. Fair often
  5. Very often
- Have you felt tense because of the fashion braces?
  1. Never
  2. Hardly ever
  3. Occasionally
  4. Fair often
  5. Very often
- Has your diet been unsatisfactory because of the fashion braces?
  1. Never
  2. Hardly ever
  3. Occasionally
  4. Fair often
  5. Very often
- Have you had to interrupt meals because of the fashion braces?
  1. Never
  2. Hardly ever
  3. Occasionally
  4. Fair often
  5. Very often
- Have you found it difficult to relax because of the fashion braces?
  1. Never
  2. Hardly ever
  3. Occasionally
  4. Fair often
  5. Very often
- Have you been a bit embarrassed because of the fashion braces?
  1. Never
  2. Hardly ever
  3. Occasionally
  4. Fair often
  5. Very often
- Have you been a bit irritable with other people because of the fashion braces?
  1. Never
  2. Hardly ever
  3. Occasionally
  4. Fair often
  5. Very often
- Have you had difficulty doing usual jobs because of the fashion braces?
  1. Never
  2. Hardly ever
  3. Occasionally
  4. Fair often
  5. Very often
- Have you felt that life in general was less satisfying because of the fashion braces?
  1. Never
  2. Hardly ever
  3. Occasionally
  4. Fair often
  5. Very often
- Have you been totally unable to function because of the fashion braces?
  1. Never
  2. Hardly ever
  3. Occasionally
  4. Fair often
  5. Very often

**Questions to the fashion braces group about oral health related quality of life.**

**How frequently have you experienced negative impacts in these dimensions during the preceding 12 months?**

- Have you had trouble pronouncing any words because of the therapeutic braces?
  1. Never
  2. Hardly ever
  3. Occasionally
  4. Fair often
  5. Very often
- Have you felt that your sense of taste has worsened because of the therapeutic braces?
  1. Never
  2. Hardly ever
  3. Occasionally
  4. Fair often
  5. Very often
- Have you had painful aching in your mouth because of the therapeutic braces?
  1. Never
  2. Hardly ever
  3. Occasionally
  4. Fair often
  5. Very often
- Have you found it uncomfortable to eat any foods because of the therapeutic braces?
  1. Never
  2. Hardly ever
  3. Occasionally
  4. Fair often
  5. Very often
- Have you been self-conscious because of the therapeutic braces?
  1. Never
  2. Hardly ever
  3. Occasionally
  4. Fair often
  5. Very often
- Have you felt tense because of the therapeutic braces?
  1. Never
  2. Hardly ever
  3. Occasionally
  4. Fair often
  5. Very often
- Has your diet been unsatisfactory because of the therapeutic braces?
  1. Never
  2. Hardly ever
  3. Occasionally
  4. Fair often
  5. Very often
- Have you had to interrupt meals because of the therapeutic braces?
  1. Never
  2. Hardly ever
  3. Occasionally
  4. Fair often
  5. Very often
- Have you found it difficult to relax because of the therapeutic braces?
  1. Never
  2. Hardly ever
  3. Occasionally
  4. Fair often
  5. Very often
- Have you been a bit embarrassed because of the therapeutic braces?
  1. Never
  2. Hardly ever
  3. Occasionally
  4. Fair often
  5. Very often
- Have you been a bit irritable with other people because of the therapeutic braces?
  1. Never
  2. Hardly ever
  3. Occasionally
  4. Fair often
  5. Very often
- Have you had difficulty doing usual jobs because of the therapeutic braces?
  1. Never
  2. Hardly ever
  3. Occasionally
  4. Fair often
  5. Very often
- Have you felt that life in general was less satisfying because of the therapeutic braces?
  1. Never
  2. Hardly ever
  3. Occasionally
  4. Fair often
  5. Very often
- Have you been totally unable to function because of the therapeutic braces?
  1. Never
  2. Hardly ever
  3. Occasionally
  4. Fair often
  5. Very often

**Questions to the control group about oral health related quality of life.**

**How frequently have you experienced negative impacts in these dimensions during the preceding 12 months?**

- Have you had trouble pronouncing any words because of problems with your mouth, teeth or gum?
  1. Never
  2. Hardly ever
  3. Occasionally
  4. Fair often
  5. Very often
- Have you felt that your sense of taste has worsened because of problems with your mouth, teeth or gum?
  1. Never
  2. Hardly ever
  3. Occasionally
  4. Fair often
  5. Very often
- Have you had painful aching in your mouth?
  1. Never
  2. Hardly ever
  3. Occasionally
  4. Fair often
  5. Very often
- Have you found it uncomfortable to eat any foods because of problems with your mouth, teeth or gum?
  1. Never
  2. Hardly ever
  3. Occasionally
  4. Fair often
  5. Very often
- Have you been self-conscious because of problems with your mouth, teeth or gum?
  1. Never
  2. Hardly ever
  3. Occasionally
  4. Fair often
  5. Very often
- Have you felt tense because of problems with your mouth, teeth or gum?
  1. Never
  2. Hardly ever
  3. Occasionally
  4. Fair often
  5. Very often
- Has your diet been unsatisfactory because of problems with your mouth, teeth or gum?
  1. Never
  2. Hardly ever
  3. Occasionally
  4. Fair often
  5. Very often
- Have you had to interrupt meals because of problems with your mouth, teeth or gum?
  1. Never
  2. Hardly ever
  3. Occasionally
  4. Fair often
  5. Very often
- Have you found it difficult to relax because of problems with your mouth, teeth or gum?
  1. Never
  2. Hardly ever
  3. Occasionally
  4. Fair often
  5. Very often
- Have you been a bit embarrassed because of problems with your mouth, teeth or gum?
  1. Never
  2. Hardly ever
  3. Occasionally
  4. Fair often
  5. Very often
- Have you been a bit irritable with other people because of problems with your mouth, teeth or gum?
  1. Never
  2. Hardly ever
  3. Occasionally
  4. Fair often
  5. Very often
- Have you had difficulty doing usual jobs because of problems with your mouth, teeth or gum?
  1. Never
  2. Hardly ever
  3. Occasionally
  4. Fair often
  5. Very often
- Have you felt that life in general was less satisfying because of problems with your mouth, teeth or gum?
  1. Never
  2. Hardly ever
  3. Occasionally
  4. Fair often
  5. Very often
- Have you been totally unable to function because of problems with your mouth, teeth or gum?
  1. Never
  2. Hardly ever
  3. Occasionally
  4. Fair often
  5. Very often

**Arabic Version Questionnaire**

**بسم الله الرحمن الرحيم**

**الرجاء قراءة هذا الاستبيان والاجابة عليه بتمعن وذلك لغايات البحث العلمي إذا كان عمرك اقل من ١٨ سنة فلا بد من الحصول على موافقة من ولي امرك. ١- نعم ٢- لا**

- كم هو عمرك؟ .....................
- ما هو الجنس؟

1. ذكر
2. أنثى

- ما هو مستواك التعليمي؟

1. الابتدائية
2. المتوسطة
3. الثانوية
4. الجامعة
5. أخرى ............

- ما هو دخل الاسرة؟

1. متوسط (حوالي ١٠ آلاف ريال)
2. اقل من المتوسط
3. اكثر من المتوسط

- هل تستخدم/ ين الدخان او الشيشة؟ ١

1. نعم
2. لا

- كم مرة تنظف /ين اسنانك في اليوم؟

1. مرة
2. مرتين
3. اقل من ذلك
4. اكثر من ذلك
5. لا افرش اسناني
6. أخرى ..................

- هل لاحظت تغير في لون الاسنان؟

1. نعم
2. لا
3. ربما

- هل ترتدي أو ترتدين تقويم الاسنان؟

1. نعم
2. لا

مجموعة تقويم الاسنان

- ما السبب لوضعك التقويم؟

1. الرغبة في تسوية وترتيب الاسنان
2. زيادة المظهر الجمالي والثقة
3. اتباع الموضة وتقليد الأصدقاء
4. التأثر بالدعايات ووسائل التواصل الاجتماعي.

- هل شعرت ان التقويم منحك مزيدا من الثقة والجمال؟

1. نعم
2. لا
3. أخرى .............

- هل لاحظت حركة او تغير في ترتيب أسنانك بعد التقويم؟

1. نعم
2. لا
3. ربما

- هل لاحظت تغير في لون الاسنان بسبب تقويم الأسنان؟

1. نعم
2. لا
3. أخرى.............

- ما نوع تقويم الاسنان الذي معك الان؟

1. علاجي
2. زينة

مجموعة تقويم الزينة

- متى قمت بتركيب التقويم سواء كان علاجي او زينة؟ عدد الشهور ..............
- اين عملت تقويم الزينة؟

1. مستشفى خاص
2. مستشفى حكومي
3. بنفسي في البيت
4. أخرى........

- إذا كان في مركز صحي فمن قام بتركيب التقويم لك؟

1. اخصائي تقويم اسنان
2. طبيب اسنان عام
3. مساعد/ة طبيب اسنان
4. لا اعلم
5. أخرى .................

- ما رأيك بسعر تقويم الزينة؟

1. مناسب جدا (رخيص)
2. مناسب الى حد ما (متوسط)
3. غير مناسب (غالي)
4. أخرى ..............

- كم كانت تكلفة تقويم الزينة؟ التكلفة الكاملة بالريال السعودي ..................
- هل تقوم بزيارة الطبيب (من وضع التقويم لك) بشكل دوري؟

1. نعم
2. لا

- اذا كانت اجابتك نعم كم مرة تزور طبيب التقويم؟

1. مرة كل شهر
2. مرة كل شهرين
3. مرة كل ثلاثة شهور
4. مرة كل أربعة شهور
5. أخرى ..............

**الأسئلة لمجموعة تقويم الزينة عن جودة الحياة المرتبطة بصحة الفم**

**كم مرة خلال العام الماضي كان لديك المشاكل ادناه في أسنانك أو لثتك بسبب تقويم الزينة؟**

1. هل كان لديك مشاكل في لفظ أي كلمة بسبب تقويم الزينة؟

- أبدا
- نادرا
- أحيانا
- معظم الوقت
- دائما

1. هل شعرت مرة أن حاسة التذوق ساءت بسبب تقويم الزينة؟

- أبدا
- نادرا
- أحيانا
- معظم الوقت
- دائما

1. هل كان لديك أي ألم في فمك بسبب تقويم الزينة؟

- أبدا
- نادرا
- أحيانا
- معظم الوقت
- دائما

1. هل شعرت مرة بصعوبة في تناول بعض المأكولات بسبب تقويم الزينة؟

- أبدا
- نادرا
- أحيانا
- معظم الوقت
- دائما

1. هل شعرت بالإحراج والقلق بأنك لست جيد المظهر لدى الأخرين؟

- أبدا
- نادرا
- أحيانا
- معظم الوقت
- دائما

1. هل شعرت مرة بالتوتر بسبب تقويم الزينة؟

- أبدا
- نادرا
- أحيانا
- معظم الوقت
- دائما

1. هل تغذيتك أصبحت غير مرضية بسبب تقويم الزينة؟

- أبدا
- نادرا
- أحيانا
- معظم الوقت
- دائما

1. هل اضطررت مرة للتوقف عن الطعام بسبب تقويم الزينة؟

- أبدا
- نادرا
- أحيانا
- معظم الوقت
- دائما

1. هل وجدت مرة صعوبة في الاسترخاء بسبب تقويم الزينة؟

- أبدا
- نادرا
- أحيانا
- معظم الوقت
- دائما

1. هل شعرت مرة بالإحراج بسبب تقويم الزينة؟

- أبدا
- نادرا
- أحيانا
- معظم الوقت
- دائما

1. هل شعرت مرة بالانزعاج مع باقي الناس بسبب تقويم الزينة؟

- أبدا
- نادرا
- أحيانا
- معظم الوقت
- دائما

1. هل وجدت مرة صعوبة في إنجاز واجباتك الاعتيادية بسبب تقويم الزينة؟

- أبدا
- نادرا
- أحيانا
- معظم الوقت
- دائما

1. هل شعرت مرة أن الحياة بشكل عام اقل إرضاء بسبب تقويم الزينة؟

- أبدا
- نادرا
- أحيانا
- معظم الوقت
- دائما

1. هل كنت مرة غير قادر كليا على العمل بسبب تقويم الزينة؟

- أبدا
- نادرا
- أحيانا
- معظم الوقت
- دائما

**الأسئلة لمجموعة تقويم الاسنان العلاجي عن جودة الحياة المرتبطة بصحة الفم**

**كم مرة خلال العام الماضي كان لديك المشاكل ادناه في أسنانك أو لثتك بسبب تقويم الاسنان؟**

1. هل كان لديك مشاكل في لفظ أي كلمة بسبب تقويم الاسنان؟

- أبدا
- نادرا
- أحيانا
- معظم الوقت
- دائما

1. هل شعرت مرة أن حاسة التذوق ساءت بسبب تقويم الاسنان؟

- أبدا
- نادرا
- أحيانا
- معظم الوقت
- دائما

1. هل كان لديك أي ألم في فمك بسبب تقويم الاسنان؟

- أبدا
- نادرا
- أحيانا
- معظم الوقت
- دائما

1. هل شعرت مرة بصعوبة في تناول بعض المأكولات بسبب تقويم الاسنان؟

- أبدا
- نادرا
- أحيانا
- معظم الوقت
- دائما

1. هل شعرت بالإحراج والقلق بأنك لست جيد المظهر لدى الأخرين؟

- أبدا
- نادرا
- أحيانا
- معظم الوقت
- دائما

1. هل شعرت مرة بالتوتر بسبب تقويم الاسنان؟

- أبدا
- نادرا
- أحيانا
- معظم الوقت
- دائما

1. هل تغذيتك أصبحت غير مرضية بسبب تقويم الاسنان؟

- أبدا
- نادرا
- أحيانا
- معظم الوقت
- دائما

1. هل اضطررت مرة للتوقف عن الطعام بسبب تقويم الاسنان؟

- أبدا
- نادرا
- أحيانا
- معظم الوقت
- دائما

1. هل وجدت مرة صعوبة في الاسترخاء بسبب تقويم الاسنان؟

- أبدا
- نادرا
- أحيانا
- معظم الوقت
- دائما

1. هل شعرت مرة بالإحراج بسبب تقويم الاسنان؟

- أبدا
- نادرا
- أحيانا
- معظم الوقت
- دائما

1. هل شعرت مرة بالانزعاج مع باقي الناس بسبب تقويم الاسنان؟

- أبدا
- نادرا
- أحيانا
- معظم الوقت
- دائما

1. هل وجدت مرة صعوبة في إنجاز واجباتك الاعتيادية بسبب تقويم الاسنان؟

- أبدا
- نادرا
- أحيانا
- معظم الوقت
- دائما

1. هل شعرت مرة أن الحياة بشكل عام اقل إرضاء بسبب تقويم الاسنان؟

- أبدا
- نادرا
- أحيانا
- معظم الوقت
- دائما

1. هل كنت مرة غير قادر كليا على العمل بسبب تقويم الاسنان؟

- أبدا
- نادرا
- أحيانا
- معظم الوقت
- دائما

**الأسئلة للمجموعة التي لم تتلقى أي نوع من تقويم الاسنان عن جودة الحياة المرتبطة بصحة الفم**

**كم مرة خلال العام الماضي كان لديك المشاكل ادناه في فمك وأسنانك ولثتك؟**

1. هل كان لديك مشاكل في لفظ أي كلمة بسبب مشاكل في فمك او أسنانك او لثتك؟

- أبدا
- نادرا
- أحيانا
- معظم الوقت
- دائما

1. هل شعرت مرة أن حاسة التذوق ساءت بسبب مشاكل في فمك او أسنانك او لثتك؟

- أبدا
- نادرا
- أحيانا
- معظم الوقت
- دائما

1. هل كان لديك أي ألم في فمك بسبب مشاكل في فمك او أسنانك او لثتك؟

- أبدا
- نادرا
- أحيانا
- معظم الوقت
- دائما

1. هل شعرت مرة بصعوبة في تناول بعض المأكولات بسبب مشاكل في فمك او أسنانك او لثتك؟

- أبدا
- نادرا
- أحيانا
- معظم الوقت
- دائما

1. هل شعرت بالإحراج والقلق بأنك لست جيد المظهر لدى الأخرين؟

- أبدا
- نادرا
- أحيانا
- معظم الوقت
- دائما

1. هل شعرت مرة بالتوتر بسبب مشاكل في فمك او أسنانك او لثتك؟

- أبدا
- نادرا
- أحيانا
- معظم الوقت
- دائما

1. هل تغذيتك أصبحت غير مرضية بسبب مشاكل في فمك او أسنانك او لثتك؟

- أبدا
- نادرا
- أحيانا
- معظم الوقت
- دائما

1. هل اضطررت مرة للتوقف عن الطعام بسبب مشاكل في فمك او أسنانك او لثتك؟

- أبدا
- نادرا
- أحيانا
- معظم الوقت
- دائما

1. هل وجدت مرة صعوبة في الاسترخاء بسبب مشاكل في فمك او أسنانك او لثتك؟

- أبدا
- نادرا
- أحيانا
- معظم الوقت
- دائما

1. هل شعرت مرة بالإحراج بسبب مشاكل في فمك او أسنانك او لثتك؟

- أبدا
- نادرا
- أحيانا
- معظم الوقت
- دائما

1. هل شعرت مرة بالانزعاج مع باقي الناس بسبب مشاكل في فمك او أسنانك او لثتك؟

- أبدا
- نادرا
- أحيانا
- معظم الوقت
- دائما

1. هل وجدت مرة صعوبة في إنجاز واجباتك الاعتيادية بسبب مشاكل في فمك او أسنانك او لثتك؟

- أبدا
- نادرا
- أحيانا
- معظم الوقت
- دائما

1. هل شعرت مرة أن الحياة بشكل عام اقل إرضاء بسبب مشاكل في فمك او أسنانك او لثتك؟

- أبدا
- نادرا
- أحيانا
- معظم الوقت
- دائما

1. هل كنت مرة غير قادر كليا على العمل بسبب مشاكل في فمك او أسنانك او لثتك؟

- أبدا
- نادرا
- أحيانا
- معظم الوقت
- دائما
